# Post-stroke Dysphagia: Prognosis and Treatment–A Systematic Review of RCT on Interventional Treatments for Dysphagia Following Subacute Stroke

**DOI:** 10.3389/fneur.2022.823189

**Published:** 2022-04-25

**Authors:** Philipp Balcerak, Sydney Corbiere, Richard Zubal, Georg Kägi

**Affiliations:** ^1^Department of Neurology and Stroke Center, Cantonal Hospital St. Gallen, St. Gallen, Switzerland; ^2^Department of Neurology, Inselspital, Bern University Hospital, and University of Bern, Bern, Switzerland

**Keywords:** swallowing, cerebrovascular, nutrition, therapy, ischemia, deglutition

## Abstract

**Purpose:**

Post-stroke dysphagia is an underdiagnosed but relevant complication, associated with worse outcome, dependency and quality of life of stroke survivors. Detailed mechanisms of post-stroke dysphagia are not very well understood, but established therapeutic concepts are needed. Different interventional studies have been published dealing with post-stroke dysphagia. This systematic review wants to collect and give an overview over the published evidence.

**Methods:**

PubMed, Embase, Cochrane, CINAHL were searched for relevant interventional studies on post-stroke dysphagia in the (sub-)acute setting (within 3 months of stroke onset). The search has been filtered for randomized trials with an inactive control and the relevant data extracted.

**Results:**

After initially finding 2,863 trials, finally 41 trials have been included. Seven different therapeutic concepts have been evaluated (Acupuncture, behavioral/physical therapy, drug therapy, neuromuscular electrical stimulation, pharyngeal electrical stimulation, transcranial direct current stimulation and repetitive transcranial magnetic stimulation). Studies of all modalities have shown some effect on post-stroke dysphagia with several studies raising concerns about the potential bias.

**Conclusion:**

The amount and quality of studies are not enough to suggest certain therapies. Some therapeutical concepts (intensive physical therapy, transcranial magnetic stimulation, drug therapy) seem to be good potential therapeutic options, but further research is needed.

## Introduction

The prevalence of dysphagia in stroke patients differs with the diagnostic method in the acute phase: 51–55% with clinical testing and 64–78% with instrumental examinations (1, 2). Impairment of swallowing still persists in up to 50% of the cases in the following course (1) and complications frequently arise. In about 40% of stroke survivors dysphagia is persistent. Patients with dysphagia have an increased risk for pneumonia which is probably linked with the severity of dysphagia since the risk is even much greater in patients with aspiration (2) and even more in patients with silent aspiration (3). Other common complications include malnutrition and dehydration, especially in the long-term (4). Malnourished and/or dehydrated stroke patients have a relevant risk of further complications and an elevated rate of mortality and dependency (5, 6). The detailed mechanisms of post-stroke dysphagia are not well understood.

Neuroanatomically different localisations of brain lesions, infra- and supratentorial, can cause dysphagia. As to frequency, brain stem lesions more often cause dysphagia compared to hemispheric strokes. Combined lesions have the highest risk for developing oropharyngeal dysphagia (7). Infratentorial lesions usually cause dysphagia through motor deficits whereas in supratentorial stroke dysphagia is usually caused by sensory-afferent deficits (8). Sensory deficits are more pronounced in dysphagic patients with aspiration.

Early recognition of the problem is linked with a better outcome. The Predictive Swallowing Score (PRESS) (9) has been developed to identify patients who are at risk for persistent dysphagia, so that treatment and potentially the placement of a percutanous enteral tube can be initiated at an early stage. Although clinical screening of dysphagia after stroke has been established routinely in several countries, instrumental screening is restricted in most. The latter has a higher diagnostic accuracy and allows a more detailed evaluation of the swallowing function, so that the problem and potentially the treatment can be adapted more specifically. FEES (10) (Flexible endoscopic evaluation of swallowing) and VFS (11) (Videofluoroscopy) are different in the procedure and in the results they provide, but allow an elaborated view of the deglutition function.

Therapeutic options comprise dietary and nutritional interventions, behavioral treatments, oral care, pharmacological- and neuro-stimulation.

Treatment guidelines contain different physical therapies and preventive measures to avoid dysphagia-associated complications, but lack medical or electrophysiological interventions to enhance dysphagia recovery after stroke in the acute or subacute setting.

The aim of this systematic review is to search the literature for published data on interventions for post-stroke dysphagia in the acute and subacute setting and to identify potential interventions and targets for further scientific research.

## Methods

The PRISMA statement (12) (Preferred Reporting Items in Systematic reviews and Meta-Analyses) has been followed throughout the process.

### Search Strategy and Selection Criteria

A systematic review of the literature was conducted to indentify all randomized cotrolled trials which assess the effect of therapeutic interventions of post-stroke dysphagia in the acute and subacute setting. The following databases were searched: Pubmed/MEDLINE, Embase, CENTRAL/Cochrane Library and The Cumulative Index to Nursing and Allied Health Literature (CINAHL). Search dates in all database were during August 2021 and consisted of the following searched terms: (^*^stroke^*^ OR cerebrovascular^*^ OR “brain ischemia”) AND (dysphagia^*^ OR “deglutition disorder” OR “impaired swallowing” OR “swallowing disorder” OR “swallowing impairment”) AND (RCT OR placebo OR “randomized controlled trial” OR double-bIind OR placebo OR “controlled clinical trial”). If possible in the database, the search was filtered using “human” and “English language”.

The reference lists of the screened articles were searched for articles which have not been included in the original search.

### Inclusions and Exclusions

All studies were included who were interventional trials about post-stroke dysphagia with a randomized controlled design. After the above-mentioned literature search duplicates were removed and titles and abstracts screened for the eligibility criteria. Here, the PICO concept has been followed. The control arm had to be an inactive comparator with all possible interventions (medication, electrical stimulation, physical therapy, etc.) included, while usual care or standard therapy were counted as eligible for this review. Dysphagia had to be assessed clinically or instrumentally prior to the start of treatment.

The treatment had to be started within 3 months after ischemic stroke. Inclusion was restricted to studies examining interventions aiming to improve mechanisms of the impaired swallowing function are examined, so that trials assessing preventive measures i.e., prophylactic antibiotic treatment or measures trying to prevent dysphagia associated complications were not included here.

Only original data/publications have been included. Comments, case series, reviews etc. were excluded, latest at full-text-screening. Literature dealing with dysphagia due to other cause than stroke or healthy individuals and their swallowing function were not included. Full-text articles in English had to be available to be included in this review.

If treatment was started after 3 months after stroke or this relevant information is missing in the data, the publication was not included. Outcome or safety outcome parameters were not part of the inclusion criteria, although publications were excluded if they did not provide dysphagia- or swallowing-specific outcomes, whereas the methodology of obtaining those (clinical, instrumental/paraclinical) was not of interest at that stage of the process. A scaling of the global swallowing function had to be provided as any outcome parameter, where all scores (already established or explained in the publication) were accepted. Studies dealing only with parts of the swallowing act i.e., only the oral transit time, were not included.

### Data Extraction

One reviewer (PB) performed the database search while two reviewers (PB, RZ) performed the screening. After removal of duplications, a title and abstract assessment was performed in a first step which was followed by a full-text screening. In case of divergent assessments, a consensus was found.

Data of the included papers were extracted into a pre-formed electronic sheet and then the trials were assessed by two reviewers (PB, SC). The data extraction followed the PICO approach: (1) Participants–stroke patients with dysphagia, (2) Interventions–any active intervention/therapy with or without the combination of conventional routine therapy, (3) Comparison–any inactive control/placebo, (4) Outcome–swallowing functions, measured instrumentally and clinically, and complications (if available). The risk of bias of every trial included was assessed according the Cochrane's Handbook with the RoB-2-tool (13). The tool was used according to the guidelines. Risk grades were high risk (+), low risk (-) and unclear risk or some concern were put together (o). The overall assessment was performed according to RoB-2-algorithm.

In case of conflicting results, a consensus was found.

## Results

### Literature Retrieved

**Figure 1:**
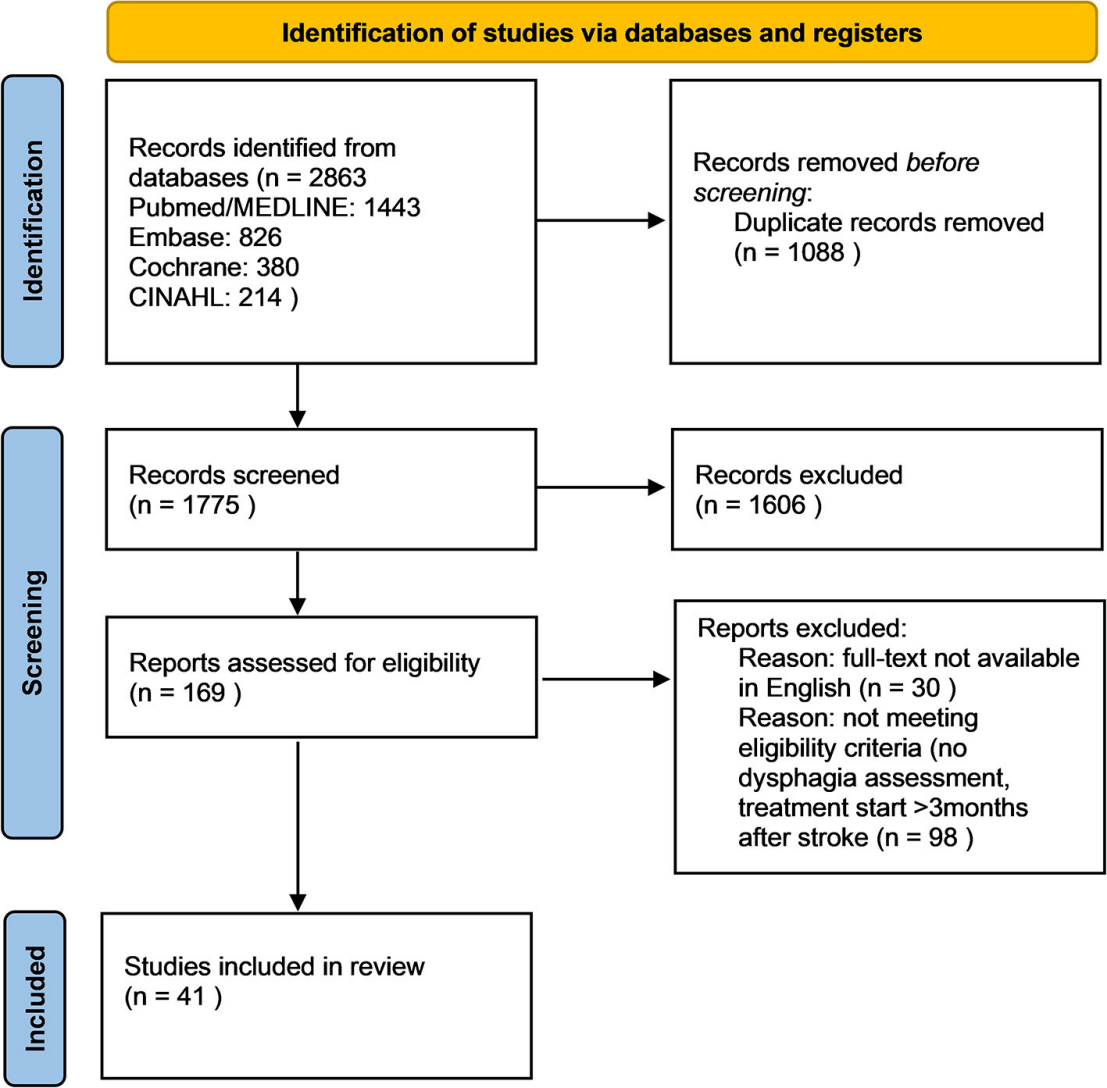
Flow diagram on literature retrieval.

The search strategy was applied and resulted in 1,367 hits with 479 duplicates, leaving 888 citations. After screening the title and abstracts 169 publications seemed eligible. After exclusion of 30 papers, which were not available in English and full-text screening for the above-mentioned eligibility criteria 41 trials were included in the review. The reasons for exclusion are mentioned after grouping in Figure 1.

### Interventions

Seven different interventional modalities have been evaluated in the trials: acupuncture in two trials, behavioral/physical therapy in 13, drug therapy in five, neuromuscular electrical stimulation (NMES) in nine, pharyngeal electrical stimulation (PES) in four, transcranial direct current stimulation (tDCS) in four and six studies assessed repetitive transcranial magnetic stimulation (two studies assessed two modalities in a three-arm design).

The start of the interventions complied with our eligibility criteria and was started within 3 months after stroke onset.

### Outcome Measures

All of the included trials had a swallowing assessment as an outcome parameter, as mentioned in the eligibility criteria. Thirty-three trials used instrumental evaluations of dysphagia as outcome assessments. For the instrumental assessments, the flexible endoscopic evaluation of swallowing (FEES) was performed in three trials, whereas videofluoroscopy (VFS) was used in 30. During both procedures different scores and scales were obtained to assess the swallowing function. Twenty-three of the included studies had clinical examinations as outcome assessments of dysphagia with different scores and scales used for the evaluation.

### Summary of Results

Forty-one trials dealing with seven different therapeutic concepts were included in this review and provided results on their effect on dysphagia recovery after stroke with a total of 2,166 participants included in these studies. Table 1 shows the study characteristics from extracted data and its findings of the single studies (Further information can be found in the Supplementary Material).

**Table 1:** Summary of results.

**References**	**Intervention *Control***	**Sample size** **(*n* =)**	**Dysphagia assessments (Scores/Techniques)**	**Summary of results**
**Acupuncture**				
Chen (14)	Acupuncture *no treatment*	250	•Bedside swallowing test •VFS	Enhanced recovery
Xia (15)	Acupuncture + usual care *Usual care*	124	•SSA •DOSS	Enhanced recovery (in the later course of follow-up)
**Behavioral/physical therapy**				
Carnaby (16)	Physical therapy in different intensity levels *usual care*	306	Paramatta Hospital's assessment of dysphagia	Intensity-level dependent Enhanced recovery
Eom (17)	Expiratory muscle strength training *Sham*	33	•VDS •PAS	Enhanced recovery
Guillen-Sola (18)^*^	Respiratory muscle strength training *Usual care (and vs. NMES)*	42 (60 in total w/ NMES)	•FOIS •PAS •DOSS	Enhance recovery (both interventional groups)
Heo (19)	Kinesio taping *No taping*	44	FDS	No difference
Hwang (20)	Tongue stretching exercises *usual care*	25	VFS	Enhanced recovery
Kim (21)	Tongue-to-palate-resistance-training	35	•VFS/DS •PAS	Improvement on VFS, but not PAS
Koyama (22)	Jaw opening exercise *isometric sham*	16	•VFS •FOIS	No difference in clinical evaluation, mixed results for instrumental scales
Li (23)	Extended and standardized behavioral and physical training (partly including acupuncture and electrical stimulation) *usual care*	40	Kubota water swallowing test	Enhanced recovery
Moon (24)	Expiratory muscle strength training *usual care*	18	•PAS •FDS	Enhanced recovery
Moon (25)	Tongue pressure strength and accuracy training *usual care*	16	MASA	Enhanced recovery
Park (26)	Effortful swallowing training *usual care*	24	VDS	Enhanced recovery
Park (27)	Jaw opening exercise *sham*	40	•PAS •FOIS	No difference
Park (28)	Chin tuck against resistance exercise *usual care*	22	•FDS •PAS	Enhanced recovery
**Drug therapy**				
Cui (29)	Oral capsaicin + ice stimulation *usual care*	92	•WST •SSA	Enhanced recovery
Feng (30)	Tongyan spray *placebo*	122	SSA	Enhanced recovery
Lee (31)	Lisinopril *placebo*	93	Royal Brisbane Hospital Outcome Measure for Swallowing	No difference
Perez (32)	Nifedipine *placebo*	17	•VFS •Clinical assessment (not specified)	Enhanced recovery
Wang (33)	Capsaicin *placebo*	69	•Volume-Viscosity Swallow Test •SSA •WST	Enhanced recovery
**Neuromuscular electrical stimulation (NMES)**				
Carnaby (34)	McNeill Dysphagia Therapy + neuromuscular electrical stimulation *McNeill Dysphagia Therapy and usual care*	53	•MASA •FOIS	Dysphagia improvement from physical therapy and NMES
Guillen-Sola (18)^*^	NMES *Usual care (and vs. respiratory muscle training)*	41 (60 in total w/ NMES)	•FOIS •PAS •DOSS	Enhance recovery (both interventional groups)
Huang (35)	NMES or NMES + usual care *usual care*	29	•FOIS •PAS •VDS	Mixed results without clear significant effect
Konecny (36)	NMES hyoid *usual care*	108	•VFS	Enhanced recovery
Lim (37)^*^	NMES *usual care (and vs. rTMS)*	33 (47 total w/ rTMS)	•PAS •FDS •ASHA NOMS	Enhanced recovery with limitations: only first follow-up, liquids and instrumental scores
Power (38)	Faucial pillar stimulation *Usual care*	16	•PAS	No difference
Umay (39)	Sensory-level electric stimulation of masseter *sham*	98	•MASA •Fiberoptic Endoscopic Dysphagia Severity Scale	Enhanced recovery
Xia (40)	NMES or NMES + usual care *usual care*	120	•VFS •SSA	Only dysphagia improvement in NMES + usual care
Lee (41)	NMES + usual care *usual care*	57	•FOIS •VFS	Enhanced recovery
**Pharyngeal electrical stimulation (PES)**				
Bath (42)	PES *sham*	162	•VFS •PAS	No difference
Dziewas (43)	PES *sham* in tracheotomized patients	69	•FEES •FOIS •dysphagia severity rating scale	Dysphagia improvement, more decannulations
Jayasekeran (44)	PES *Sham*	28	•VFS/PAS	Enhanced recovery
Vasant (45)	PES *sham*	36	•Dysphagia severity rating scale •PAS	No difference
**Transcranial direct current stimulatiuon (tDCS)**				
Kumar (46)	tDCS *sham*	14	•DOSS	Enhanced recovery
Pingue (47)	tDCS *sham*	40	•DOSS	No difference
Suntrup-Krueger (48)	tDCS *sham*	60	•Fiberoptic Endoscopic Dysphagia Severity Scale	Enhanced recovery
Yang (49)	tDCS *sham*	16	•FDS	Enhanced recovery
**Repetitive Transcranial magnetic stimulation (rTMS)**				
Du (50)	rTMS high- and low-intensity *sham*	40	•SSA •WST •Degree of dysphagia	Enhanced recovery
Khedr (51)	rTMS *sham*	22	•WST •Degree of dysphagia	Enhanced recovery
Lim (37)^*^	rTMS *usual care*	29 (47 total w/ NMES)	•PAS •FDS •ASHA NOMS	Enhanced recovery with limitations: only first follow-up, liquids and instrumental scores
Tarameshlu (52)	rTMS + usual care and rTMS *usual care*	18	•MASA •FOIS	Enhanced recovery only for combined therapy
Khedr (53)	rTMS *sham*	26	•WST •Degree of dysphagia	Enhanced recovery
Park (54)	Bilateral rTMS, unilateral rTMS *sham*	35	•Clinical Dysphagia Scale •DOSS •PAS •VFS/VDS	Enhanced recovery only for bilateral rTMS

*Summary of study characteristics. Summary of results show the statistical results as to the rejection of H_0_. If VFS or FEES are mentioned, specific algorithm for detailed evaluation of the swallowing function have been established. VFS, Videofluoroscopy; VDS, Videofluoroscopic dysphagia scale; PAS, Penetration Aspiration Scale; FDS, Functional Dysphagia Scale; FOIS, Functional oral intake scale; MASA, Mann Assessment of Swallowing Ability; WST, water swallowing test; SSA, standardized swallowing assessment; DOSS, Dysphagia Outcome and Severity Scale; ASHA NOMS, American Speech-Language Hearing Association National Outcomes Measurements System (swallowing scale used).*

* *These studies used three arms with usual care being one of those, that is why these studies are listed in two interventional groups*.

#### Acupuncture

Two studies (14, 15) were included which compared Acupuncture vs. no specific therapy or standard care. In one study (14), patients with hemiplegic stroke were included with swallowing outcome on instrumental testing (videofluoroscopy) as a secondary endpoint, so that only a subgroup of the initial sample size was included. Acupuncture showed some effect on post stroke dysphagia within the first 7 weeks after stroke onset. In the other trial (15), the intervention showed some effect, but only in the later course of the trial, which lasted only 4 weeks.

#### Behavioral/Physical Therapies

Thirteen trials (16–28) have been included which assess the impact physical training and/or the intensity (15, 19) of physical therapy on dysphagia recovery. Three studies (17, 18, 24) evaluated forced respiratory muscle training against resistance and its effect on post-stroke dysphagia, where all could show enhanced dysphagia recovery. Among the other trials, two of them assess jaw opening exercises (22, 27), while the other concepts had similar aspects, but different maneuvers used in their studies (for details see Table 1, or Supplementary Material). The assessments differed within the studies, so that three trials (16, 23, 25) only used clinical measurements for dysphagia evaluation. In total, nine trials showed positive impact of the therapies on dysphagia recovery, whereas two (19, 27) did not show any difference and two other (21, 22) showed mixed results in different dysphagia assessments. Out of the nine studies showing positive impact, two (20, 26) only had impact on the oral phase of the swallowing act.

#### Drug Therapy

Four different medication classes have been assessed as to their effect on dysphagia rehabilitation. Caspaicin was used in two trials (29, 33), nifedipine (32), lisinopril (31) and tongyan spray (30) in one each. All studies except lisinopril showed significant differences as to recovery of the swallowing function.

#### Neuromuscular Electrical Stimulation

In the big majority of included trials (18, 34–41), a therapeutical effect on post-stroke dysphagia could have been shown, with one (35) having mixed results and one (38) showing no difference, though. Five of the studies (18, 34, 35, 40, 41) assessed the addition of NMES to standard or physical therapy. The combination of NMES and standard therapy has been shown to be more effective than NMES alone in the comparative studies. Whereas, most studies used stimulation of hyoid muscles, two (38, 39) used different stimulation locations. The stimulation of faucial pillars (38) failed to show a significant improvement over standard therapy. The sensory-level stimulation of the masseter muscle (39) seems to have a bigger benefit over standard care on dysphagia recovery.

#### Pharyngeal Electrical Stimulation

Of the four studies (42–45) in this review, two (43, 44) reached the study goal and showed a positive effect on post-stroke dysphagia. One study (43) included was in a further specified study population with stroke survivors needing tracheotomy.

#### Transcranial Direct Current Stimulation

In this modality group, all trials (46–49) used instrumental dysphagia assessments, but not structured clinical scales. All of them used sham procedures as control groups, but had different concepts of stimulation. Two studies (46, 48) implemented anodal tDCS over the unaffected hemisphere, one study (49) used anodal tDCS over the affected motor cortex and all achieved to show a positive effect. The other trial (47) had a dual concept with anodal stimulation over the lesioned and cathodal over the contralesional side. This failed to show an enhanced dysphagia rehabilitation.

#### Repetitive Transcranial Magnetic Stimulation

All of the six included trials (37, 50–54) showed at least some therapeutic effect of rTMS on post-stroke dysphagia. The concept of applying the stimulation over the affected motor cortex using the hotspotting technique was similar in the included trials. The frequency varied between 1 Hz up to 10 Hz. Tarameshlu (52) used three groups with one rTMS alone, rTMS and standard therapy combined and standard therapy only as the control, where only the combined therapy showed a significant effect. Park (54) compared bi- and unilateral TMS to a sham procedure where only the bilateral stimulation showed a significant effect.

### Risk of Bias

Every trial was evaluated for risk of bias applying the RoB-2-tool. The results can be found in Table 2 grouped by interventions performed in the trials. Since mentioned in the inclusion criteria, all studies were randomized and were compared to an inactive control group.

**Table 2:** Summary of risk of bias assessment.

**Risk of bias domains**	**Randomization**	**Deviation from intervention**	**Missing outcome data**	**Outcome measurements**	**Reporting bias**	**Overall bias**
**Acupuncture**					
Chen (14)	-	+	+	-	-	+
Xia (15)	O	+	-	-	-	+
**Behavioral/physical therapy**					
Carnaby (16)	-	+	-	-	-	+
Eom (17)	O	-	O	-	-	O
Guillen-Sola (18)^*^	-	-	O	-	-	O
Heo (19)	O	O	-	+	-	+
Hwang (20)	-	+	+	-	-	+
Kim (21)	O	+	O	-	-	+
Koyama (22)	-	O	+	-	-	+
Li (23)	+	+	-	o	-	+
Moon (24)	O	O	-	-	-	O
Moon (25)	-	+	+	0	-	+
Park (26)	-	+	+	-	-	+
Park (27)	-	+	-	-	-	+
Park (28)	-	+	O	-	-	+
**Drug therapy**					
Cui (29)	-	+	+	O	-	+
Feng (30)	-	O	-	O	-	O
Lee (31)	-	-	+	-	-	+
Perez (32)	-	-	-	-	-	-
Wang (33)	-	-	O	-	-	O
**Neuromuscular electrical stimulation (NMES)**					
Carnaby (34)	-	+	-	-	-	+
Guillen-Sola (18)^*^	-	O	O	-	-	O
Huang (35)	O	+	-	-	-	+
Konecny (36)	O	+	-	-	-	+
Lim (37)^*^	O	O	O	-	-	+
Power (38)	O	O	O	-	-	+
Umay (39)	-	O	O	-	-	O
Xia (40)	O	O	-	-	-	O
Lee (41)	-	O	-	-	-	O
**Pharyngeal electrical stimulation (PES)**					
Bath (42)	O	O	-	-	-	O
Dziewas (43)	-	-	+	-	-	+
Jayasekeran (44)	-	O	-	-	-	-
Vasant (45)	-	-	-	-	-	-
**Transcranial direct current stimulatiuon (tDCS)**					
Kumar (46)	-	O	O	-	-	O
Pingue (47)	-	O	-	-	-	-
Suntrup-Krueger (48)	-	-	-	-	-	-
Yang (49)	O	O	-	-	-	O
**Repetitive transcranial magnetic stimulation (rTMS)**					
Du (50)	-	-	-	-	-	-
Khedr (51)	O	O	-	-	-	O
Lim (37)^*^	O	O	O	-	-	+
Tarameshlu (52)	-	O	-	-	-	O
Khedr (53)	O	-	-	-	-	O
Park (54)	O	O	-	-	-	O

*Risk of Bias assessment. Overview over consensus result of the risk of bias assessment, using the risk-of-bias-tool-2 according to Cochrane's handbook. – low risk, 0 some concerns or unclear risk, + high risk. Overall risk assessment according to RoB-2-algorithm.*

* *These studies used three arms with usual care being one of those. That is why these studies are listed in two interventional groups*.

Overall, in several studies data for the assessment of bias was missing, so that the risk of bias could not be assessed properly (graded as 0 = some concerns / unclear bias). Other studies were evaluated as high risk (+) of bias which was majorly in the blinding (of the therapy and the outcome assessments) as well as the allocation or randomization process. Some studies were missing relevant outcome data. No relevant concerns were raised as to the reporting bias. Overall bias was performed according to the RoB-2-algorithms with an overall high risk with one domain being at high risk and low risk with all domains being at low risk. Results in between were evaluated according to the assessors' judgement.

All of the studies assessing behavioral or physical therapy and NMES were assessed overall as at least “some concerns”, with a vast majority due to concerns as to the deviation from intervention, to a major part because of blinding issues. The PES and tDCS group of trials were the only ones with the a relevant part of studies being assessed as low-risk (Detailed results in Table 2).

## Discussion

This systematic review gives an overview over the RCTs in the field of therapeutic interventions for post-stroke dysphagia. In this review, especially the (sub-)acute phase was of interest, so that only trials assessing therapeutic modalities, which have started within 3 months after stroke were included.

In total, 41 RCTs were included with seven different therapeutic concepts.

As to acupuncture, two studies were included, which showed an effect on post-stroke dysphagia. It has to be mentioned, though, that in one study (14), only a subgroup of these 250 participants received distinct dysphagia assessments and were therefore included in the statistical analyses. Together with the high risk of bias for both acupuncture studies, results of the study have to be interpreted cautiously. Having this in mind the European guidelines (55) give a weak recommendation that acupuncture may be used in the rehabilitation of swallowing function.

Most studies assess behavioral and physical options, but are at high risk of bias, so that conclusions cannot be made based on these results definitively. Additionally, most studies examine similar, but different kind of therapeutic maneuvers. It seems that the intensity of swallowing therapy has a positive effect on dysphagia recovery and should be a safe procedure. As to the specification of therapy, this review cannot give a clear answer. Respiratory muscle strength training seems to give a positive effect on post-stroke dysphagia based on the three studies (17, 18, 24) in this review. Otherwise, different kind of tongue strengthening interventions were observed with mixed result as to oropharyngeal dysphagia, since some showed only effect on the oral phase (without effect on the pharyngeal phase). Some studies only used clinical scores which are known to be less sensitive than instrumental ones. Jaw opening exercises failed to show an effect (for details see Table 1 or Supplementary Material). If any, respiratory muscle strength training seem to be safe and potentially beneficial choice, but due to the high risk of bias the evidence has to be improved in this area. Nevertheless, a patient tailored behavioral therapy can be recommended.

As yet, no concept of drug therapy has been established, so that four different drug classes have been used. The mechanisms and concepts of drug therapy in the context of post stroke dysphagia mainly goes into the direction of increasing pharyngeal levels of substance P. This can be achieved by stimulating TRPV1 and TRPM8 (56, 57) agonists. Even ACE inhibitors are also believed to increase substance P (58). All drugs except Lisinopril have shown promising results with most of them leading to concerns about the risk of bias. Due to the lack of large clinical trials and systematic instrumental dysphagia assessments, Nifedipine and Capsaicin (TRPV1 agonist) are substances of interest but can't be recommended for clinical routine so far. Nevertheless, further studies with these substances to evaluate their clinical impact on clinical outcome measures in stroke patients are needed and should be performed.

The majority of studies about neurostimulation techniques in dysphagia are superficial stimulation (NMES), tDCS, rTMS and PES. The latter three endeavor to change neuroplasticity of specific brain areas. In theory, this can include facilitation of a lesioned brain region or facilitation of healthy brain areas. In case of post stroke dysphagia, the interventions have the goal to facilitate brain regions involved in swallowing through increasing synaptic efficiency and cortical reorganization. Functional imaging studies (59, 60) succeeded to show that the primary motor cortex seems to play a major role in the act of volitional swallowing and can be associated with post-stroke dysphagia (61).

The studies about tDCS in the rehabilitation of post stroke dysphagia can't be specified further as to the localization and mode of stimulation. Two out of the four studies used the anodal stimulation over the unaffected hemisphere and did show some effect. Another one stimulated the affected side and showed improved swallowing rehabilitation, too. The other study used a dual stimulation concept without the proof of benefit. These concepts have to be confirmed in further studies given the very low number of RCTs included in this review.

Studies investigating rTMS in the rehabilitation of post stroke dysphagia show also a fair methodological quality leading to some concerns of bias. All show some effect on post-stroke dysphagia. It does not come as a surprise that the trials with combined rTMS and standard therapy have better effects, neither that bilateral stimulation seems to have a more pronounced therapeutical impact. Different aspects of the swallowing function seem to be represented in different hemispheres and different parts of those (62). Furthermore, studies with instrumental and clinical testing of dysphagia assessment are broadly lacking because most of those included in this review have used either clinical or instrumental dysphagia assessments only.

The studies assessing PES are of comparably low risk of bias, but only two did show some effect on post-stroke dysphagia. This effect was only seen in one study in a subgroup of tracheotomized stroke survivors, which seemed to profit from the stimulation therapy.

The trials on neurostimulation all show some methodological weaknesses, leading to concerns about the risk of bias. Some effect, especially in combination with standard therapy or additional physical therapy has been shown and is promising. Nevertheless, this has to be proven in further high-quality research with clinical endpoints. The ESO guidelines (55) give a weak recommendation on the adjunct use of neurostimulation in post stroke dysphagia.

This review covers most of the relevant databases. The search strategy is quite selective due to restricting to RCT with inactive controls, allowing only interventions targeting the swallowing function and therefore leaving out preventive, dietary or nutritional measures. The selected start of the intervention within 3 months of stroke focused on the acute/subacute phase. Therefore, there could be a lack of some therapeutic concepts for post-stroke dysphagia, but we believe that it gives a comprehensive overview over the existing publications of RCT in order to help to enhance further scientific research and develop therapies.

It becomes clear, that the field of dysphagia needs more research to establish therapeutic guidelines. According to the findings above it seems safe and reasonable to say that an intensive swallowing or respiratory muscle strength therapy should be applied with rTMS and some drug candidates as potential future options for additional therapeutical concepts. It has to be stated that main outcome measurements for primary endpoints should be instrumental in addition to clinical ones, whenever feasible.

## Data Availability Statement

The original contributions presented in the study are included in the article/Supplementary Material, further inquiries can be directed to the corresponding author.

## Author Contributions

All authors listed have made a substantial, direct, and intellectual contribution to the work and approved it for publication.

## Funding

This work was supported from internal resources of the Department of Neurology and Stroke Center, Cantonal Hospital St. Gallen, St. Gallen, Switzerland.

## Conflict of Interest

The authors declare that the research was conducted in the absence of any commercial or financial relationships that could be construed as a potential conflict of interest.

## Publisher's Note

All claims expressed in this article are solely those of the authors and do not necessarily represent those of their affiliated organizations, or those of the publisher, the editors and the reviewers. Any product that may be evaluated in this article, or claim that may be made by its manufacturer, is not guaranteed or endorsed by the publisher.
