# Supplementary material for: Post-stroke Dysphagia: Prognosis and Treatment–A Systematic Review of RCT on Interventional Treatments for Dysphagia Following Subacute Stroke
Source: Front Neurol. 2022 Apr 25;13:823189. doi: 10.3389/fneur.2022.823189 (PMC9082350; doi:10.3389/fneur.2022.823189)
Supplement: Supplementary file 1 [file Table_1.DOC]

Supplementary Material

**Table.** Details of included studies (methodology and risk of bias assessments), sorted by therapeutical modality

| **Acupuncture** | |
| --- | --- |
| ***Chen (2016)*** | |
| **Intervention**  **Sample size**  **Outcome measurements**  **Result** | Acupuncture vs. no therapy  n = 250 in total, only 133 dysphagic patients analyzed with 125 included in statistical analysis  Clinical: Bedside swallowing test  Instrumental: VFS  Enhanced dysphagia recovery |
| **RoB assessment** | Participants and some caregivers not blinded  Some outcome data missing |
| ***Xia (2016)*** |  |
| **Intervention**  **Sample size**  **Outcome measurements**  **Result** | Acupuncture + usual care vs. usual care  124  Clinical: standardized swallowing assessment  Instrumental: dysphagia outcome severity scale  Enhanced dysphagia recovery at last visit (not before) |
| **RoB assessment** | Missing information on randomization / allocation  Participants and caregivers not blinded |
|  | |
| **Behavioural/physical therapy** | |
| ***Carnaby (2006)*** | |
| **Intervention**  **Sample size**  **Outcome measurements**  **Result** | Usual care vs. low-intensity vs. high-intensity swallowing therapy  Therapies consisted of:   - Low-intensity: compensation strategies, environmental changes (upright position i.e.), swallowing advice and dietary modifications, 3 times per week - High-intensity: direct swallowing exercises, dietary modifications daily (or every working days in outpatient setting   n = 306  Clinical: Paramatta Hospital’s assessment of dysphagia  Instrumental: none  High-intensity therapy showed marked enhancement of dysphagia recovery |
| **RoB assessment** | Patients and primary caregivers (SLP) were not blinded |
| ***Eom (2017)*** | |
| **Intervention**  **Sample size**  **Outcome measurements**  **Result** | Expiratory muscle strength training vs. sham  n = 33  Instrumental: VDS, PAS  Enhanced recovery showed in both scales used |
| **RoB assessment** | Missing information on treatment allocation  Relevant outcome data missing |
| ***Guillen-Sola (2016)*** | |
| **Intervention**  **Sample size**  **Outcome measurements**  **Result** | Expiratory muscle strength training + usual care vs. NMES + sham training + usual care vs. usual care  n = 62  Clinical: FOIS  Instrumental: PAS, DOSS  Enhanced recovery showed in all scales used |
| **RoB assessment** | Relevant drop-outs / outcome data missing  (no blinding procedures for NMES group) |
| ***Heo (2015)*** |  |
| **Intervention**  **Sample size**  **Outcome measurements**  **Result** | Kinesio taping vs. no taping  n = 44  Instrumental: VFS  No significant difference between groups as to dysphagia assessment (secondary outcome), primary outcome muscle excursion with significant difference of unclear clinical value |
| **RoB assessment** | Some concerns on randomization  Patients and caregivers were not blinded  Outcome measurements used are in doubt of answering the question about a dysphagia treatment |
| ***Hwang (2019)*** | |
| **Intervention**  **Sample size**  **Outcome measurements**  **Result** | Standardized tongue stretching exercises vs. usual care  n = 25  Instrumental: VFS  Enhanced recovery of oral, but not pharyngeal deglutition phase |
| **RoB assessment** | Participants and caregivers were not blinded  Some outcome data missing |
| ***Kim (2017)*** | |
| **Intervention**  **Sample size**  **Outcome measurements**  **Result** | Standardized tongue-to-palate-resistance training vs. usual care  n = 35  Instrumental: VFS with dysphagia score and Penetration Aspiration Scale  No improvement of PAS, but for VDS |
| **RoB assessment** | Some information of randomization missing  Participants and caregivers not blinded  Relevant part of outcome data missing |
| ***Koyama (2017)*** | |
| **Intervention**  **Sample size**  **Outcome measurements**  **Result** | Modified jaw opening exercises (with biofeedback) vs. sham procedure  n = 16  Clinial: FOIS  Instrumental: VFS  No difference in clinical measurements, with mixed results for different phases of the instrumental assessments |
| **RoB assessment** | Blinding issues  approx. ¼ drop-outs |
| ***Li (2017)*** | |
| **Intervention**  **Sample size**  **Outcome measurements**  **Result** | Extended / intensified training (incl. ice stimulation and active physical swallowing training) vs. usual care (which comprises different types of acupuncture and electrotherapy, etc.)  n = 40  Clinical: Kubota water swallowing test  Instrumental: None  Enhanced recovery |
| **RoB assessment** | No information on randomization  No blinding of participants, caregivers nor assessors  Only clinical evaluation |
| ***Moon (2017)*** |  |
| **Intervention**  **Sample size**  **Outcome measurements**  **Result** | Expiratory muscle training vs. usual care  18  Instrumental: FDS and PAS  Enhanced recovery in all scales |
| **RoB assessment** | Missing information on randomization  Participants and caregivers were not blinded |
| ***Moon (2018)*** | |
| **Intervention**  **Sample size**  **Outcome measurements**  **Result** | Standardized tongue pressure exercise vs. usual care  n = 16  Clinical: Mann’s Assessment of Swallowing Ability  Instrumental: None  Enhanced recovery in clinical scores |
| **RoB assessment** | Participants and caregivers were not blinded  Relevant part of outcome data missing |
| ***Park (2019)*** | |
| **Intervention**  **Sample size**  **Outcome measurements**  **Result** | Effortful swallowing training (Active tongue strength training) vs. usual care  n = 24  Instrumental: VFS  Enhanced recovery of oral, but not pharyngeal phase |
| **RoB assessment** | Participants and some caregivers not blinded  Outcome data missing, drop-outs amongst others probably due to intervention-related issues |
| ***Park (2020)*** | |
| **Intervention**  **Sample size**  **Outcome measurements**  **Result** | Resistive jaw opening vs. no therapy / usual care  n = 40  Clinical: FOIS  Instrumental: PAS  No between group differences, in both groups dysphagia improved |
| **RoB assessment** | Participants, caregivers and assessors not blinded / or missing information |
| ***Park (2018)*** | |
| **Intervention**  **Sample size**  **Outcome measurements**  **Result** | Chin tuck resistance training vs. no therapy / usual care  n = 22  Clinical: Functional dysphagia scale  Instrumental: PAS  Enhanced recovery |
| **RoB assessment** | Some concerns on outcome data missing |
|  | |
| **Drug therapy** | |
| ***Cui (2020)*** | |
| **Intervention**  **Sample size**  **Outcome measurements**  **Result** | Capsaicin vs. standard care (incl. ice stimulation in both groups)  n = 92  Clinical: Water swallowing test, standardized swallowing assessment  Instrumental: none  Enhanced recovery |
| **RoB assessment** | Participants and caregivers not blinded  Missing outcome data due to medical reasons, potentially intervention-associated  Clinical outcome measurements |
| ***Feng (2012)*** | |
| **Intervention**  **Sample size**  **Outcome measurements**  **Result** | Tongyan spray vs. no therapy  n = 122  Clinical: standardized swallowing assessment  Instrumental: none  Enhanced recovery |
| **RoB assessment** | No information on blinding  Clinical assessments only |
| ***Lee (2015)*** | |
| **Intervention**  **Sample size**  **Outcome measurements**  **Result** | Lisinopril 2.5 mg vs. placebo  n = 93  Clinical: Royal Brisbane Hospital Outcome Measure for Swallowing  Instrumental: none  No between group difference |
| **RoB assessment** | Relevant outcome data missing |
| ***Perez (1998)*** | |
| **Intervention**  **Sample size**  **Outcome measurements**  **Result** | Nifedipin 30mg/d vs. placebo  n = 17  Clinical: Clinical assessment (not specified)  Instrumental: VFS  Enhanced recovery of clinical and instrumental assessments |
| **RoB assessment** | No real bias issues, although clinical assessment is not specified |
| ***Wang (2019)*** | |
| **Intervention**  **Sample size**  **Outcome measurements**  **Result** | Capsaicin vs. placebo  n = 69  Clinical: Volume-Viscosity Swallow Test, standardized swallowing assessment, water swallowing test  Instrumental: none  Enhanced recovery of clinical assessments |
| **RoB assessment** | Some concerns on outcome data, but not probably linked to intervention  (Only clinical assessments) |
|  | |
| **Neuromuscular electrical stimulation** | |
| ***Carnaby (2020)*** | |
| **Intervention**  **Sample size**  **Outcome measurements**  **Result** | NMES (VitaStim) + Active physical Dysphagia Therapy (McNeill Dysphagia Therapy) vs. MDTP and usual care  n = 53  Clinical: FOIS, MASA  Instrumental: none  Enhanced recovery from NMES + physical therapy (MDTP) |
| **RoB assessment** | Blinding issues especially or the physical dysphagia therapy |
| ***Guillen-Sola (2016)*** | |
| **Intervention**  **Sample size**  **Outcome measurements**  **Result** | Expiratory muscle strength training + usual care vs. NMES + sham training + usual care vs. usual care  n = 62  Clinical: FOIS  Instrumental: PAS, DOSS  Enhanced recovery showed in all scales used |
| **RoB assessment** | Relevant drop-outs / outcome data missing  no blinding procedures for NMES group |
| ***Huang (2014)*** | |
| **Intervention**  **Sample size**  **Outcome measurements**  **Result** | NMES (VitalStim) vs. NMES + usual care vs. usual care  n = 29  Clinical: FOIS  Instrumental: PAS, VDS  Mixed results for PAS and VDS, but always without reaching significance level |
| **RoB assessment** | Missing information on randomization  Participants and caregivers not blinded |
| ***Konecny (2018)*** | |
| **Intervention**  **Sample size**  **Outcome measurements**  **Result** | NMES (TENS on hyoid muscles) vs. usual care  n = 108 33  Instrumental: VDS  Enhanced recovery |
| **RoB assessment** | No information on randomization and blinding procedures |
| ***Lim (2014)*** | |
| **Intervention**  **Sample size**  **Outcome measurements**  **Result** | NMES (VitalStim on hyoid muscles) vs. usual care (vs. rTMS)  n = 33 (47 in total w/ rTMS)  Clinical: ASHA NOMS (swallowing scale)  Instrumental: PAS, FDS  In general mixed results: enhanced recovery after 2 weeks (not significant after 4 weeks), only for liquid consistencies and only for instrumental measures. No difference for clinical outcomes |
| **RoB assessment** | Missing information on allocation  Participants and caregivers were not blinded  Relevant outcome data missing out (drop-outs) |
| ***Power (2006)*** | |
| **Intervention**  **Sample size**  **Outcome measurements**  **Result** | Anterior faucial pillar stimulation 0.2Hz vs. sham  n = 16  Instrumental: PAS  No difference between groups |
| **RoB assessment** | Missing information on allocation, randomization, blinding procedures, drop-outs / missing data |
| ***Umay (2017)*** | |
| **Intervention**  **Sample size**  **Outcome measurements**  **Result** | Sensory-level electric stimulation of masseter muscle vs. sham procedure  n = 98  Clinical: MASA  Instrumental: Fiberoptic Endoscopic Dysphagia Severity Scale  Enhanced recovery of clinical and instrumental scores |
| **RoB assessment** | Missing information on blinding procedures and concerns about adequate sham procedure  Missing information on missing data, drop-outs |
| ***Xia (2011)*** | |
| **Intervention**  **Sample size**  **Outcome measurements**  **Result** | NMES + usual care vs. NMES vs. usual care  n = 120  Clinical: standardized swallowing assessment  Instrumental: VDS  Enhanced recovery only in NMES + usual care group |
| **RoB assessment** | No information on randomization nor blinding |
| ***Lee (2014)*** | |
| **Intervention**  **Sample size**  **Outcome measurements**  **Result** | NMES + usual care vs. usual care  n = 57  Clinical: FOIS  Instrumental: VFS  Enhanced recovery only in NMES + usual care group |
| **RoB assessment** | Missing information on blinding procedures |
|  | |
| **Pharyngeal electrical stimulation** | |
| ***Bath (2016)*** | |
| **Intervention**  **Sample size**  **Outcome measurements**  **Result** | PES vs. sham (PES 5Hz at threshold + 75%)  n = 162  Instrumental: VFS, PAS  No between group difference |
| **RoB assessment** | Relevant amount of outcome data missing  Some blinding issues for caregivers |
| ***Dziewas (2018)*** | |
| **Intervention**  **Sample size**  **Outcome measurements**  **Result** | PES vs. sham  n = 69 (only tracheotomised patients)  Clinical: FOIS  Instrumental: FEDSS  Enhanced recovery, more/earlier decannulations |
| **RoB assessment** | Some blinding issues for caregivers  Some concerns on outcome data missing |
| ***Jayasekeran (2010)*** | |
| **Intervention**  **Sample size**  **Outcome measurements**  **Result** | PES vs. sham (PES 5Hz at threshold + 75%)  n = 28  Instrumental: VFS, PAS  Enhanced dysphagia recovery (no PA-scores compared, but percentage of swallows with PAS > 3) |
| **RoB assessment** | Information missing on allocation concealment and blinding |
| ***Vasant (2016)*** | |
| **Intervention**  **Sample size**  **Outcome measurements**  **Result** | PES vs. sham  n = 69 (only tracheotomised patients)  Clinical: Dysphagia severity rating scale  Instrumental: PAS  No between group differences |
| **RoB assessment** | No relevant concerns of bias |
|  | |
| **Transcranial direct current stimulation** | |
| ***Kumar (2011)*** | |
| **Intervention**  **Sample size**  **Outcome measurements**  **Result** | Anodal tDCS vs. sham in unaffected hemisphere (inferior sensorimotor cortex and premotor brain regions – C3/T3-C4/T4)  n = 14  Instrumental: DOSS  Enhanced recovery |
| **RoB assessment** | Simple randomization  Missing information on outcome data |
| ***Pingue (2018)*** | |
| **Intervention**  **Sample size**  **Outcome measurements**  **Result** | Anodal tDCS over lesioned cathodal over the unaffected hemisphere vs. sham  n = 40  Instrumental: DOSS  No between group difference |
| **RoB assessment** | Some blinding concerns of caregivers with unclear risk of bias |
| ***Suntrup-Krueger (2018)*** | |
| **Intervention**  **Sample size**  **Outcome measurements**  **Result** | Anodal tDCS (over motor cortex of the unaffected side) vs. sham  n = 60  Instrumental: FEDSS  Enhanced recovery |
| **RoB assessment** | Some concerns of caregivers not blinded |
| ***Yang (2012)*** | |
| **Intervention**  **Sample size**  **Outcome measurements**  **Result** | Anodal tDCS (over pharyngeal motor cortex of the affected side) vs. sham  n = 16  Instrumental: FDS  Enhanced recovery at last follow-up (after 3 months); other measurements (different transit times) without significant differences |
| **RoB assessment** | Missing information about allocation, randomization and blinding |
|  |  |
|  | |
| **Repetitive transcranial magnetic stimulation** | |
| ***Du (2016)*** | |
| **Intervention**  **Sample size**  **Outcome measurements**  **Result** | rTMS high frequency (3Hz) vs low frequency (1Hz) vs. sham over hot spot  n = 40  Clinical: standardized swallowing assessment, water swallowing test, degree of dysphagia  Instrumental: none  Enhanced recovery |
| **RoB assessment** | No relevant concerns of bias |
| ***Khedr (2010)*** | |
| **Intervention**  **Sample size**  **Outcome measurements**  **Result** | rTMS 3Hz over affected motor cortex vs. sham  n = 22  Clinical: water swallowing test, degree of dysphagia  Instrumental: none  Enhanced recovery |
| **RoB assessment** | missing information on some parts of allocation and blinding process |
| ***Lim (2014)*** | |
| **Intervention**  **Sample size**  **Outcome measurements**  **Result** | rTMS 1Hz over pharyngeal motor cortex vs. usual care (vs. NMES)  n = 29 (47 in total w/ NMES)  Clinical: ASHA NOMS (swallowing scale)  Instrumental: PAS, FDS  In general mixed results: enhanced recovery after 2 weeks (not significant after 4 weeks), only for liquid consistencies and only for instrumental measures. No difference for clinical outcomes |
| **RoB assessment** | Missing information on allocation  Participants and caregivers were not blinded  Relevant outcome data missing out (drop-outs) |
|  |  |
| ***Tarameshlu (2019)*** | |
| **Intervention**  **Sample size**  **Outcome measurements**  **Result** | low frequency 1Hz rTMS (over “hot spot of mylohyoid muscles”) vs. usual care vs. combination  n = 18  Clinical: MASA, FOIS  Instrumental: none  Enhanced recovery only for combined therapy |
| **RoB assessment** | some concerns about blinding especially for rTMS (no sham) |
| ***Khedr (2009)*** | |
| **Intervention**  **Sample size**  **Outcome measurements**  **Result** | rTMS affected motor cortex vs. sham  n = 26  Clinical: water swallowing test, degree of dysphagia  Instrumental: none  Enhanced recovery |
| **RoB assessment** | randomization unclear |
| ***Park (2017)*** | |
| **Intervention**  **Sample size**  **Outcome measurements**  **Result** | bilateral 10 Hz vs. unilateral 10 Hz rTMS vs. Sham  n = 35  Clinical: clinical dysphagia scale  Instrumental: VFS/VDS, DOSS, PAS  Enhanced recovery only for bilateral rTMS |
| **RoB assessment** | missing information on blinding and allocation sequences |
